# Object stiffness recognition and vibratory feedback without *ad-hoc* sensing on the Hannes prosthesis: A machine learning approach

**DOI:** 10.3389/fnins.2023.1078846

**Published:** 2023-02-16

**Authors:** Giulia Bruni, Andrea Marinelli, Anna Bucchieri, Nicolò Boccardo, Giulia Caserta, Dario Di Domenico, Giacinto Barresi, Astrid Florio, Michele Canepa, Federico Tessari, Matteo Laffranchi, Lorenzo De Michieli

**Affiliations:** ^1^Rehab Technologies, Istituto Italiano di Tecnologia, Genoa, Italy; ^2^Department of Informatics, Bioengineering, Robotics System Engineering (DIBRIS), University of Genova, Genoa, Italy; ^3^Department of Electronics, Information and Bioengineering (NearLab), Politecnico of Milan, Milan, Italy; ^4^The Open University Affiliated Research Centre at Istituto Italiano di Tecnologia (ARC@IIT), Genoa, Italy; ^5^Department of Electronics and Telecommunications, Politecnico of Torino, Turin, Italy; ^6^Newman Laboratory, Massachusetts Institute of Technology, Boston, MA, United States

**Keywords:** closed-loop control, stiffness recognition, vibrotactile feedback, vibromotor, Hannes prosthetic hand, non-linear logistic regression

## Abstract

**Introduction:**

In recent years, hand prostheses achieved relevant improvements in term of both motor and functional recovery. However, the rate of devices abandonment, also due to their poor embodiment, is still high. The embodiment defines the integration of an external object – in this case a prosthetic device – into the body scheme of an individual. One of the limiting factors causing lack of embodiment is the absence of a direct interaction between user and environment. Many studies focused on the extraction of tactile information *via* custom electronic skin technologies coupled with dedicated haptic feedback, though increasing the complexity of the prosthetic system. Contrary wise, this paper stems from the authors' preliminary works on multi-body prosthetic hand modeling and the identification of possible intrinsic information to assess object stiffness during interaction.

**Methods:**

Based on these initial findings, this work presents the design, implementation and clinical validation of a novel real-time stiffness detection strategy, without *ad-hoc* sensing, based on a Non-linear Logistic Regression (NLR) classifier. This exploits the minimum grasp information available from an under-sensorized and under-actuated myoelectric prosthetic hand, Hannes. The NLR algorithm takes as input motor-side current, encoder position, and reference position of the hand and provides as output a classification of the grasped object (no-object, rigid object, and soft object). This information is then transmitted to the user *via* vibratory feedback to close the loop between user control and prosthesis interaction. This implementation was validated through a user study conducted both on able bodied subjects and amputees.

**Results:**

The classifier achieved excellent performance in terms of F1Score (94.93%). Further, the able-bodied subjects and amputees were able to successfully detect the objects' stiffness with a F1Score of 94.08% and 86.41%, respectively, by using our proposed feedback strategy. This strategy allowed amputees to quickly recognize the objects' stiffness (response time of 2.82 s), indicating high intuitiveness, and it was overall appreciated as demonstrated by the questionnaire. Furthermore, an embodiment improvement was also obtained as highlighted by the proprioceptive drift toward the prosthesis (0.7 cm).

## 1. Introduction

Upper limb loss is a serious impairment due to its explicit and direct interaction with the external world. To compensate for this loss, prostheses have been introduced to restore the functionality of human limbs during activities of daily living (ADLs). This necessity led to the development of high-tech devices with multiple degrees of freedom (Medynski and Rattray, 2011; Van Der Niet and Van Der Sluis, 2013), capable of performing a variety of gestures and grasps. However, the embodiment of these devices into the human body scheme and their acceptance are also essential elements for reconnection with the outside world (Cuberovic et al., 2019; Castellini, 2020). The term “Embodiment” means the integration of an external object in the internal corporal scheme as if it was part of the body itself. In this specific context, the external object is, precisely, the prosthesis (Longo et al., 2008). Embodiment comprises three correlated factors, namely, ownership, localization, and agency (Stiegelmar et al., 2020), and it has been suggested to promote intuitive control, learning, and comfort when using new tools, thus providing the opportunity to improve the user interface for devices such as artificial limbs. The introduction of direct feedback modalities can prevent amputees to rely exclusively on sight (Biddiss et al., 2007; Pylatiuk et al., 2007), reducing the mental effort and, therefore, facilitating the communication between user intention and prosthesis action (Markovic et al., 2018; Valle et al., 2018; Clemente et al., 2019). In fact, it has been demonstrated that the introduction of haptic feedback improves the control of the prosthesis (Mayer et al., 2020; Sensinger and Dosen, 2020; Yildiz et al., 2020; Chai et al., 2022) due to its fundamental role during human–objects interactions (Hsiao et al., 2011; Valle et al., 2018; Pena et al., 2019; Di Pino et al., 2020; Shehata et al., 2020; Raspopovic et al., 2021), allowing subjects to embody the device (Antfolk et al., 2013; Svensson et al., 2017; Raspopovic et al., 2021), hence, improving the compliance among the user, the prosthesis, and the grasped objects (Osborn et al., 2016). In the literature, this interaction is mainly assessed by providing grasp force or proprioceptive information (Stephens-Fripp et al., 2018). Contrarily, the aim of this study is to deliver information about the grasped object's stiffness that in normal conditions, occurs due to the combination of visual sensory information, proprioceptive sensations related to shape and size, and tactile sensations related to stiffness (Garland and Miles, 1997). Therefore, the current research activity offers an intuitive, non-invasive, and easy-to-use prosthetic system capable of identifying simple grasped object proprieties when visual sensory information of the user is not available or limited (Sensinger and Dosen, 2020). For instance, when the user is taking an object from a bag without looking at it or when the light in the environment is off. This situation was also treated by the Cybathlon 2020 competition, which introduced the Haptic Box task, considering it as a common ADL (Caserta et al., 2022).

Focusing on tactile sensations, several studies tried to reproduce the properties of human skin endowing the device with tactile sensing technologies that typically requires cumbersome add-on like sensing skin with different kinds of sensors such as piezoresistive (Osborn et al., 2018), capacitive (Cannata et al., 2008), piezoelectric (Yi and Zhang, 2016), and also optical (Zhao et al., 2016). The measurements acquired by these tactile sensors are often given as input to machine learning algorithms, which extract useful information that may be conveyed to the prosthesis users, as described by Jamali and Sammut (2011); Liarokapis et al. (2015); Konstantinova et al. (2017); Devaraja et al. (2020); Huang and Rosendo (2022).

Once the tactile information has been extracted, it is necessary to effectively provide it to the subject. The sensory substitution process can be exploited non-invasively, involving the connection of a certain event with specific feedback that is not the natural one, such as tactile sensory feedback (Clemente et al., 2015; Dosen et al., 2016; Patel et al., 2016; Štrbac et al., 2016; Sensinger and Dosen, 2020). For example, the subject can be taught to associate a certain vibratory stimulus with the contact of the prosthesis with an object (Antfolk et al., 2012b; Clemente et al., 2015, 2019; Dosen et al., 2016; Štrbac et al., 2016; De Nunzio et al., 2017; Nemah et al., 2020; Mamidanna et al., 2021). In contrast, superficial stimulation could target portions of the missing limb's skin that are innervated by afferent neurons after the amputation, the so-called referred touch, to stimulate the phantom limb and improve the embodiment (Antfolk et al., 2013; Masteller et al., 2021), such as kinesthetic sensory feedback.

The most common feedback restoration method is through vibration (Masteller et al., 2021), given its compatibility with electromyography (EMG) control and better acceptance by the subjects with respect to electrostimulation, capable of stimulating phantom limb sensation with electric surface charge (Shannon, 1976; Kaczmarek et al., 1991; Vargas et al., 2019). It is possible to provide different types of information acting on the amplitude and frequency of the vibration, as exploited in the study of Witteveen et al. (2013), in which the magnitude of the grasp force was transmitted using different levels of amplitude. An alternative to this feedback is the mechanotactile, as proposed using tactors by Meek et al. (1989), producing a one-to-one correspondence of touch sensation to user stimulation, or with a cuff, as proposed by Casini et al. (2015).

However, despite the high potentiality offered by these solutions, they are mainly bulky and heavy, and difficult to integrate, along with high-power consumption due to high computational burden. An example is proposed by Antfolk et al. (2012a) who designed a touch sensory feedback *via* air-mediated pressure from the hand to the forearm skin. This is a no-power solution that has neither impact on power consumption nor on computational burden. However, the final integration within the prosthesis does not guarantee the anthropomorphism of the hand device. It is also important to point out that the quick disconnection between the socket and the hand prosthesis is lost due to the mechanical connections running from the fingers' hand to the on-socket actuators. Standard devices use an electronic slip ring combined with a quick disconnect mechanism integrated into the prono-supinator wrist to guarantee the overall disconnection of the hand prosthesis from the socket in case of emergency. However, in the proposed design, this feature is compromised. Other examples are Oddo et al. (2016) and Shehata et al. (2018) who proposed an artificial fingertip to improve the performance of prosthetic hands by using intraneural stimulation. That solution can be nicely integrated into a fingertip by maintaining the anthropomorphic characteristics. However, the on-board electronics that record, process the tactile information, and encode the stimulation are cumbersome. Moreover, the high-power consumption of the FPGA-based solution does not permit the entire system to last for an entire day and to fit into a standard socket. Similarly, Clemente et al. (2019) developed a solution whose electronic skin offers high sensitivity ranging from light touch to heavy touch. However, a similar integration problem of the dedicated board occurs. In contrast, Vargas et al. (2021) finally proposed force and position sensors on the fingers to provide object stiffness recognition on amputees through vibrotactile feedback. That solution can be easily integrated; however, the performances of such a solution are limited in comparison with our results. Due to these issues, the lack of a suitable feedback restitution method in the prosthetic field is still far from being solved. Two other solutions for object stiffness recognition, without dedicated sensors, were implemented by Balasubramanian et al. (2021) and Wang et al. (2021). Their studies demonstrated the feasibility of these approaches in a robotic scenario using an actuated mechanical gripper.

Considering the advantages of providing feedback to amputees to improve the comfort between the user and the device, in this study, we first investigated the possibility of detecting void grasp and object grasp. Then, we identify the softness and hardness of the objects, therefore, permitting the user to discriminate among “void grasp,” “rigid object,” and “soft object” without visual sensory information. In the first preliminary study (Bruni and Bucchieri, 2021), a virtual multi-body model of Hannes was developed to offline demonstrate, with a virtual simulation, how the motor-side current absorption and the position measurement could be correlated with the hand grasp force and the grasped object's stiffness. Subsequently, in the following study (Bruni et al., 2022), an Ensemble Bagged Trees classifier was implemented and offline tested with simulated data to validate an approach to distinguish two different objects' stiffness.

Consequently, in the present article, we exploited the previously preliminary validated approach to develop an online (real-time) solution to perform object stiffness recognition and sensory feedback. The performance of this solution was assessed on end-users, both able-bodied and amputees. A non-linear logistic regression (NLR) classifier was used to recognize rigid or soft objects and void grasps. We excluded embedded force sensors, whose introduction would require facing many challenges, starting from the choice of the right sensor with basic requirements like high resolution, high sensitivity, and robustness, to the difficulties of managing the wiring (Kappassov et al., 2015). Instead, we proposed a methodology that uses *intrinsic sensors* (sensors and parameters already available on the prosthesis) for the normal functionality of the prosthesis that does not increase the cost and complexity of the device. In particular, we exploited the following intrinsic sensors: the motor-side current, whose relationship with the contact stiffness has been analytically demonstrated by Deng et al. (2020); the reference position, given as input to control the device closure; and the position effectively measured by the encoder (encoder position). We implemented a closed-loop vibratory feedback, using a single vibromotor embedded in the Hannes system, closely related to the predictions made by the classifier. In detail, we applied the strategy of strong vibration for rigid objects and small vibration for soft objects, which was identified in this study as “Two Feedback (2FB) condition” (Cipriani et al., 2011; Tejeiro et al., 2012). In the first phase, the classifier performance and the 2FB effectiveness were evaluated with 18 able-bodied subjects by measuring the classification accuracy through F1Score. In the second phase, a comparison between our proposed feedback method (2FB) and three other control feedback conditions was carried out on five amputees. This comparison was performed both objectively by measuring F1Score, users' response time, and proprioceptive drift, and subjectively through the questionnaire to investigate the users' appreciation of the feedback strategies and identify the most intuitive and effective one.

## 2. Materials and methods

### 2.1. Subjects

A total of 18 able-bodied subjects aged between 24 and 50 years (28.8 ± 6.2) and 5 mono-lateral amputees (right transradial amputees and users of active prostheses) were recruited for this study, with the definition described in Table 1. Written informed consent was obtained from all the subjects. The experimental protocol was approved by the Area Vasta Emilia Centro (AVEC) Ethics Committee (Protocol Code: CP-PPRAS1/1-03) and performed in accordance with the guidelines of the Declaration of Helsinki.

**Table 1:** Population of amputees.

**Amputees**	**Age**	**Time from amputation**	**Dominant limb (before amputation)**	**Amputated limb**	**Etiology**	**Level of amputation**	**Type of prosthesis**
A1	53	32 years	Right	Right	Work accident	Unilateral medial	Michelangelo hand
A2	42	18 years	Right	Right	Car accident	Unilateral proximal	Variplus hand
A3	58	37 years	Right	Right	Work accident	Unilateral distal	Michelangelo hand
A4	35	12 years	Right	Right	Work accident	Unilateral distal	Variplus hand
A5	68	53 years	Right	Right	Work accident	Unilateral distal	Michelangelo hand

### 2.2. Experimental setup

**Figure 1:**
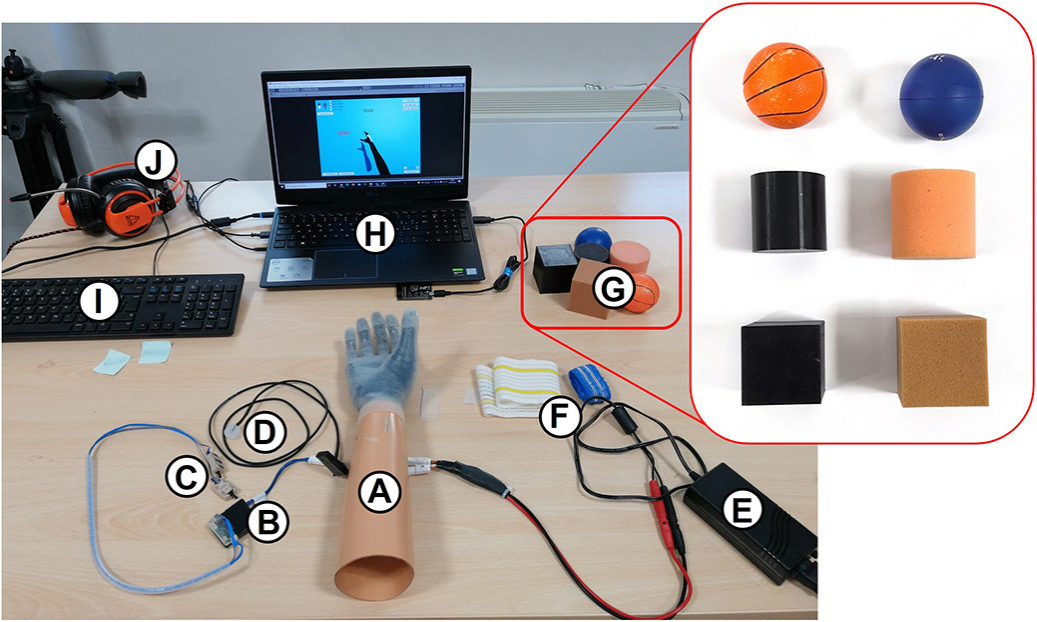
Experimental setup used to perform the task on healthy subjects and amputees. It consists of: **(A)** socket with the Hannes prosthesis; **(B)** the EMGM to control the Hannes hand, recognize the grasped object, and provide feedback; **(C)** EMG sensors; **(D)** vibromotors; **(E)** power supply; **(F)** elastic band to attach the EMGs and vibromotors to the forearm; **(G)** the objects used to perform the task; **(H)** laptop with the virtual reality; **(I)** keyboard to choose between rigid, soft, or void; **(J)** headphones to isolate the participants during the experiment.

The experimental setup that used for performing the entire experiment (Figure 1) was composed of (A) the myoelectric prosthesis Hannes, fixed on a rigid cone; (B) a custom master-board to control the hand, decode the stiffness of the grasped object, and communicate with the PC *via* Bluetooth; (C) two EMG sensors (standard Ottobock, 13E200 = 50 AC) to close or open the hand; (D) an eccentric rotating mass (ERM) vibromotor to convey the feedback; (E) a power supply for the prosthetic system; (F) two wristbands to attach the EMG sensors and the vibromotor to the subject's forearm; (G) three rigid objects and three soft objects with spherical, cubic, and cylindrical shape used during the Cybathlon 2020 edition (Medynski and Rattray, 2011; Caserta et al., 2022); (H) a laptop to choose the feedback condition and to collect the data; (I) a keyboard, placed in front of the subject, to press the left (rigid object) and right (soft object) arrows to indicate the guessed stiffness of the grasped object; and (J) headphones reproducing white noise to prevent the users from hearing the prosthesis motor.

The vibromotor was inserted into a custom silicone holder to localize and absorb the radiating stimulation and to avoid the possible heating of the skin due to prolonged vibration. The vibromotor was placed vertically with respect to the skin to produce a stronger and more focused sensation. The vibration frequency was set to 200 Hz, using a supply voltage of 2.5 V (Vybronics, 2021)^1^, and the amplitude was varied through pulse width modulation (PWM).

#### 2.2.1. The Hannes hand

Hannes is an under-actuated poly-articulated prosthetic hand characterized by a leader-follower wire configuration used to control the movements of fingers (Laffranchi et al., 2020). The hand powertrain consists of a single DC motor coupled with a custom planetary gearhead, which drives the grasping movement (refer to Supplementary material). The actuation system is controlled by a position reference (ϑ_ref_) synthesized from the user's EMG signals. A magnetic encoder measures the slow shaft position (ϑ_out_) of the hand drive train, therefore, controlling the desired grasp configuration. The low-level control system is based on a series of proportional–integrative–derivative (PID) controllers. The outer loop is position based (where only proportional and derivative (PD) terms are deployed), while the inner loop is current based and concerns proportional and integrative (PI) terms only. In particular, the error (ε_pos_) between ϑ_ref_ (hand control command) and ϑ_out_ (outer feedback) is fed to the outer PD loop. The related output is then multiplied by a proportional gain, resulting in a current reference (i_ref_) which is subtracted with the measured one (i_out_, inner secondary feedback which is the current absorbed by the DC motor during hand movement and grasp). As consequence, the related error (ε_i_) is then fed to the inner PI controller, hence, generating the control command (V) to be delivered to the motor driver. As with many under-actuated prostheses, Hannes is under-sensorized. Indeed, the only available measurements are motor-side current and position.

#### 2.2.2. Feedback conditions

**Figure 2:**
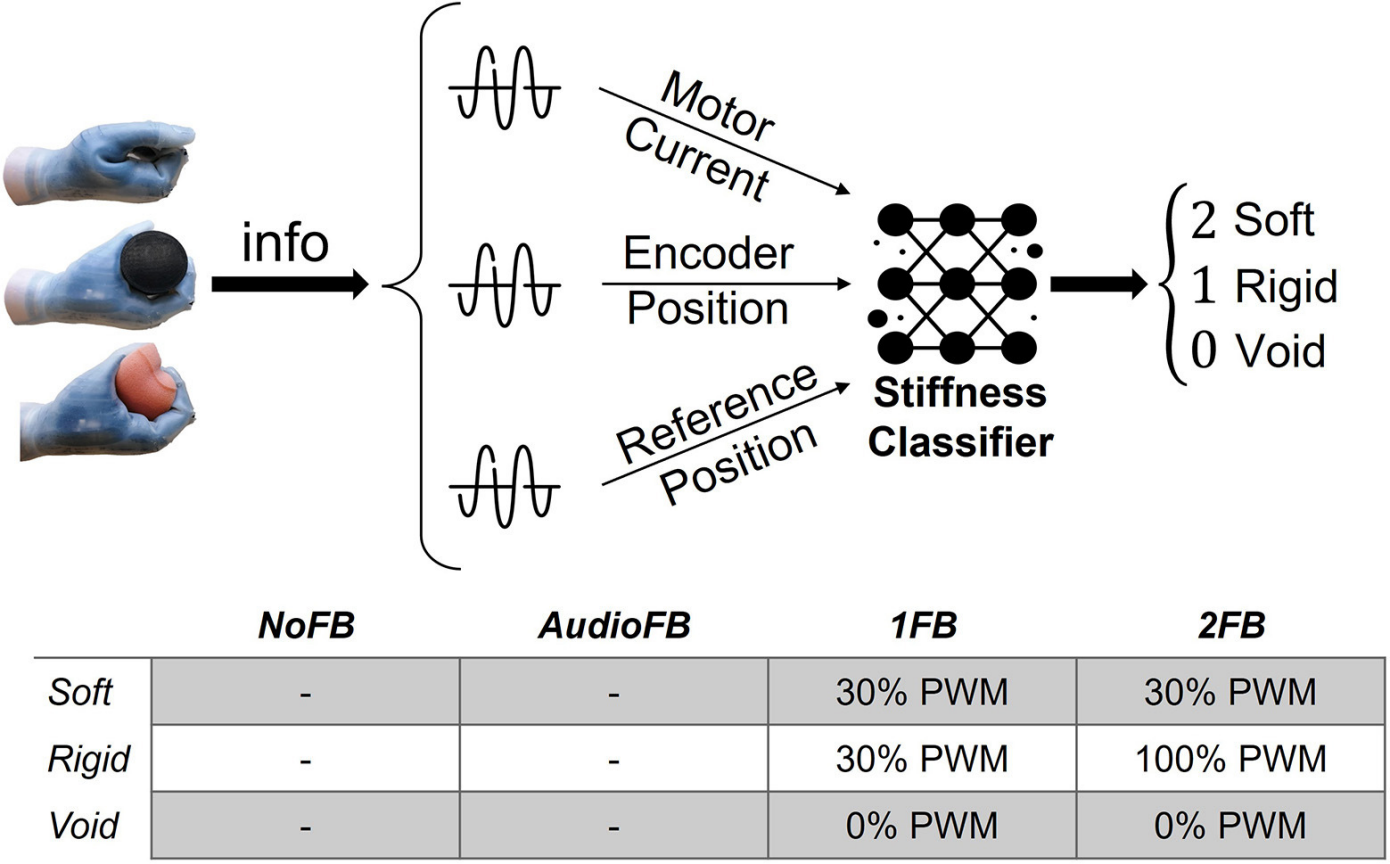
Scheme representing the object stiffness classification process. The motor-side current, the reference, and the encoder positions are acquired from Hannes and sent as input to the classifier, which gives as output the resulting stiffness.

The following four different feedback conditions were assessed in this study: (i) no FB condition (NoFB); (ii) audio FB condition (AFB); (iii) one FB condition (1FB); and (iv) two FB conditions (2FB). The NoFB condition was characterized by the absence of any possible feedback. The subjects were visibly (with closed eyes) and auditorily (headphones with white noise) blind and without any vibratory feedback. In the AFB condition, no vibratory feedback was supplied to the user, but the absence of the headphones permitted accidental auditory feedback of the moving prosthesis. In the 1FB condition, the vibratory feedback was provided, but the same vibration intensity (30% of PWM) was associated with both rigid and soft objects, while no vibration was given during void closures (refer to the table in Figure 2). The 2FB condition provided a strong vibration for rigid objects (100% of PWM) and a light vibration for soft objects (30% of PWM, a value found during some previous pilot tests to be perceived sufficiently different from the 100% used for rigid objects; refer to the table in Figure 2). As in the 1FB condition, void closures did not provide any kind of vibration. The no FB condition was implemented as a baseline for validation and comparison of subjects' performance. In fact, in the total absence of feedback, subjects' performance should be close to a random guess. The audio FB condition was introduced, since it represents a reasonable scenario of the use of the prosthetic hand by amputees, namely with no direct vision of the prosthesis but accidental auditory information from the prosthesis motion. Therefore, this second condition works as a real-case scenario ground truth for the user. The other two conditions, i.e., 1FB and 2FB, were implemented to observe, respectively, if additional vibratory feedback could improve the stiffness estimation performance, and if a different degree of vibration could further help amputees in discerning between harder and softer objects.

### 2.3. Non-linear logistic regression

#### 2.3.1. Algorithm model

The algorithm chosen for the object stiffness discrimination task is the NLR classifier. This machine learning algorithm was selected given the good performance shown for multiclass classification problems, and for simplicity reasons, since the NLR is already employed for the Hannes pattern recognition control strategy (Marinelli et al., 2020; Di Domenico et al., 2021). It is based on the calculation of the class membership probability through the following formulation:


$$\begin{array}{l} P\left(1|x,\vartheta \right)=\;\{\begin{array}{c} g\left(\vartheta^{T}\cdot x\right)=\frac{1}{1+e^{-\left(\vartheta^{T}\cdot x+\vartheta_{0}\right)}}\\ 1-P\left(y=0|x,\vartheta \right) \end{array} \end{array}$$

Where ϑ and ϑ_0_ are the internal parameters vector of the classifier and the bias term, respectively; *x* is the input feature vector, while g(·) is the sigmoid logistic function. The class prediction is obtained from the comparison between the distribution *P*(*y*|*x*) with a decision threshold (TH) as:


$$\begin{array}{l} h_{\vartheta }\left(x\right)=\{\begin{array}{c} P\left(1|x,\;\vartheta \;\right)\geq TH\to 1\\ P\left(1|x,\;\vartheta \;\right)\leq TH\to 0 \end{array} \end{array}$$

The TH value was obtained after an optimization phase on the validation set. Since the NLR is a binary classifier, a One-vs-All approach was implemented to address the multiclass classification problem for the discrimination between rigid, soft, and void closures. This involves the use of as many binary classifiers as the classes for prediction, and each of them is trained to recognize the specific class. The model parameters (ϑ) are the result of an optimization process that involves the minimization of a cost function called cross-entropy error J:


$$\begin{array}{l} J\left(\vartheta ,\vartheta_{0}\right)=\;-\frac{1}{m}\cdot \left[\overset{m}{\underset{i=1}{\sum }}y\left(i\right)\cdot \ln\left(g\left(\vartheta^{T}\cdot x+\vartheta_{0}\right)\right)\right]\\ -\frac{1}{m}\cdot \left[\overset{m}{\underset{i=1}{\sum }}\left(1-y^{\left(i\right)}\right)\cdot \ln\left(1-g\left(\vartheta^{T}\cdot x+\vartheta_{0}\right)\right)\right]\; \end{array}$$

Where *m* is the number of samples used to train the algorithm and *y*(*i*) is the known class membership of the i_th_ sample (Dellacasa Bellingegni et al., 2017; Marinelli et al., 2020).

#### 2.3.2. Algorithm training

**Figure 3:**
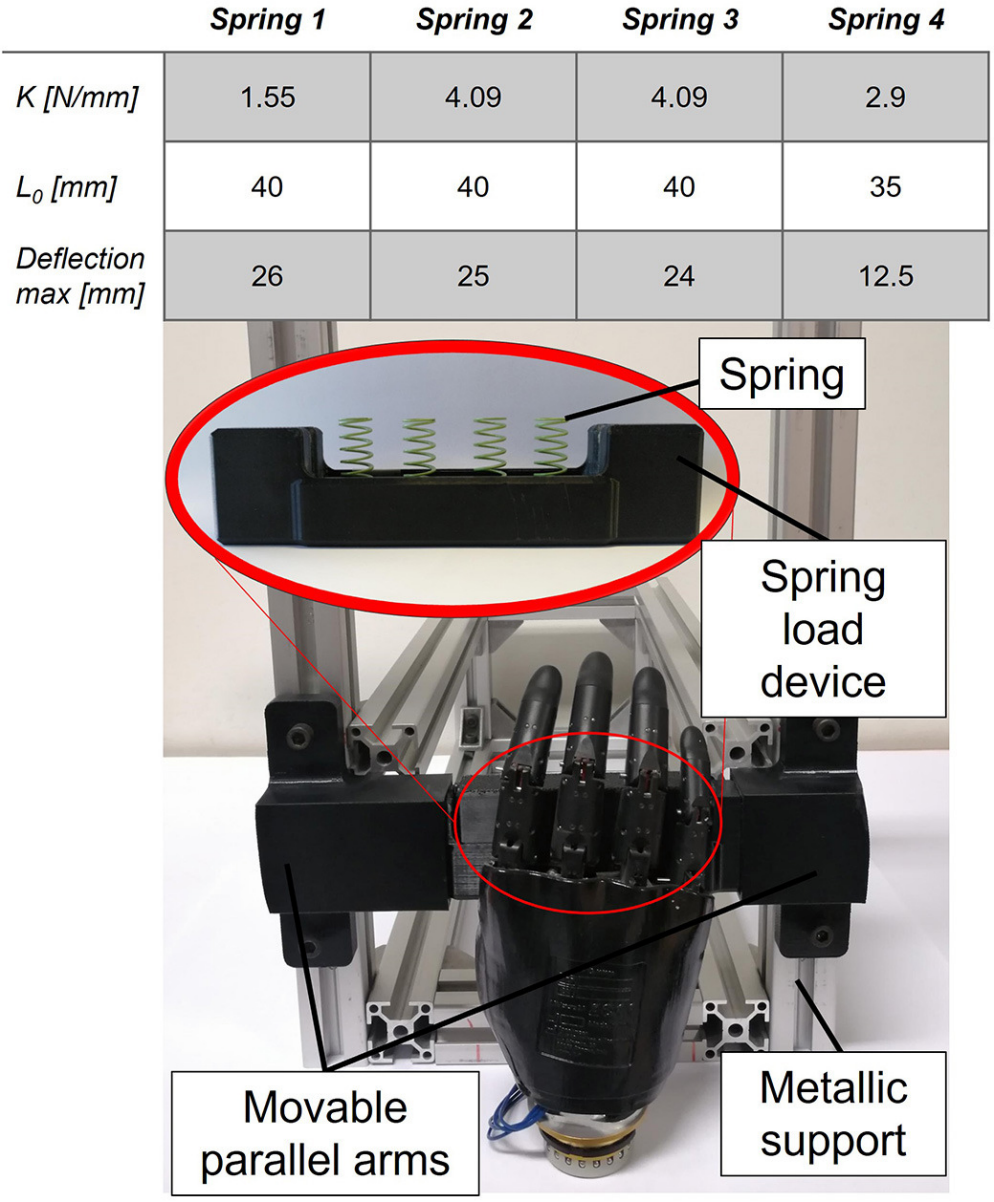
Custom-made hand dynamometer mounted on test bench used for the classifier training. The device is composed of a base where at maximum four strings can be inserted, and a cover which can slide along the base when compressed by the prosthesis fingers. The test bench consists of a custom-made system composed by movable parallel arms and a two-cuff system acting as holder. The table represents the different spring force and levels to characterize the stiffness of the dynamometer.

To adapt the model to distinguish multiple rigidities, the classifier required a training phase involving the repetitive closure of the prosthesis on objects of different stiffness. To simplify this work and to create a reproducible acquisition setup, a custom-made object was 3D printed. This device, as shown in Figure 3, was designed to reproduce the same shape and dimension of the Go Direct^®^ Hand Dynamometer (Vernier, 2021), used in the previous study (Bruni and Bucchieri, 2021), which offers the possibility to insert springs of different stiffness, simulating the grasping of soft and rigid objects, as shown in the table of Figure 3.

The device was mounted in an *ad-hoc* designed test bench. It was composed of movable parallel arms and a two-cuff system acting as a holder. The prosthesis was fixed at the base of this test bench, as shown in Figure 3, in such a way that only the distal phalanges of the four fingers had an impact on the upper plate of the device when performing a closure.

Hannes was controlled through a USB GUI, which allowed the data acquisition (motor-side current and encoder position) as well.

The NLR model generation was performed offline through MATLAB and it required training and test datasets, both characterized by the following four-column structure: (i) the motor-side current, (ii) the reference position sent as input, (iii) the encoder position measured, and (iv) the labels of the objects (rigid, soft, and void), as it is a supervised learning algorithm. All these measurements are fed to the classifier as analog signals; thus, they are directly used as the input dataset. Moreover, the label zero was associated with void closures (for motor-side currents, below 300 mA), one to the rigid objects and two to the soft objects. The dataset was created using the test bench described in Figure 3.

The choice of relying on only the motor current and the reference and measured motor encoder position was based on the immediate and relevant available sensor information on the prosthesis. Specifically, the motor current is proportional to the motor torque and, thus, to the grasp force, while the encoder position is related to the grasping motion of the fingers. In addition, the difference between reference and measured encoder position provides good information regarding the distinction between a void closure and the actual grasping of an object (this is due to the variation between the reference encoder positions that continues to grow due to EMG residuals, while the actual measured encoder position stops when encountering an object during grasp). These three quantities (current, reference, and measured positions) represent, according to the authors, the minimum set of variables to properly classify the different types of grasping (refer to the “Results” section for details on the performances). Nonetheless, it is worth mentioning that additional sensors or derived quantities could be beneficial for a more complex classifier structure. For example, motor speed, if not particularly noisy or delayed, could help in more advanced classification algorithms.

To generate the variability of the data, multiple grasps with various stiffness were performed by the prosthesis, which was controlled by both EMG and sinusoidal references. The hand dynamometer was used to simulate rigid objects, while four types of springs with distinct stiffness were used to reproduce a range of softness/soft objects, as shown in the table of Figure 3. The springs are placed under a bar to distribute the stiffness of their combination to the entire grasp. The chosen combination of springs is different for each case because the total stiffness of parallel springs varies according to their sum, thus affecting the total grasp behavior. In particular, several closures were performed for each case, as described in Table 2, to collect data for the training and validation of the NLR model. The training dataset was split into a training set (80%), used for the model generation (selection of the best model parameters (ϑ) by minimizing the cost function J), and a validation set (20%) to find the best threshold (TH). Lastly, the classifier was evaluated on the test dataset.

**Table 2:** Dataset realization for training the NLR for object stiffness recognition algorithm.

**Number closures**	**Grasped object**	**Stiffness**	**Control signal**
10	Void	Void	Sinusoidal
10	Hand dynam	Rigid	Sinusoidal
10	Hand dynam	Rigid	EMG
5	4xS1	Soft	Sinusoidal
5	4xS1	Soft	EMG
5	2xS1	Soft	Sinusoidal
5	2xS1	Soft	EMG
5	4xS2	Rigid	Sinusoidal
5	4xS2	Rigid	EMG
5	4xS3	Rigid	Sinusoidal
5	4xS3	Rigid	EMG
5	2xS1–2xS4	Soft	Sinusoidal
5	2xS1–2xS4	Soft	EMG

### 2.4. Experimental protocol

**Figure 4:**
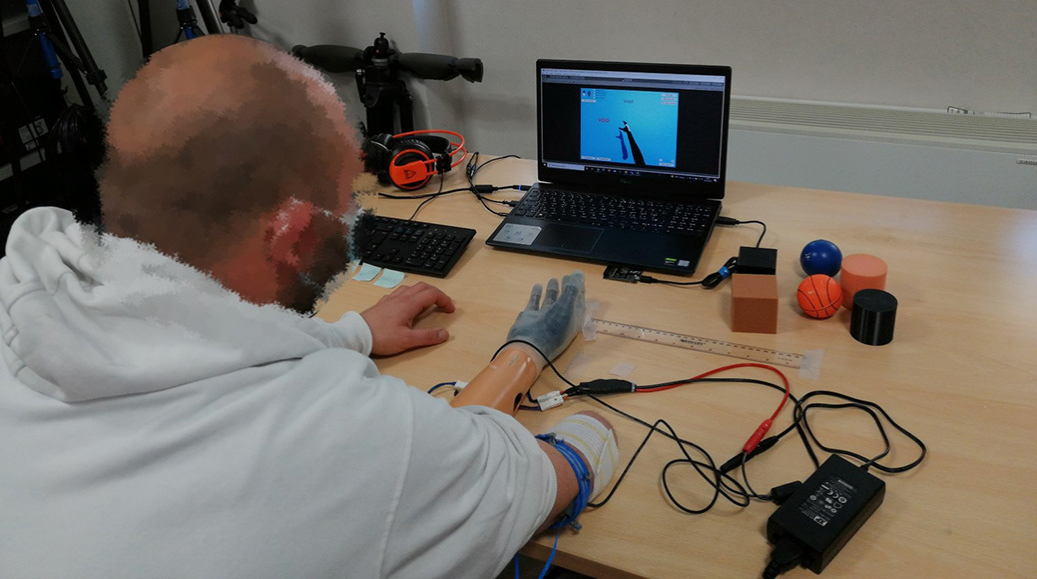
Example of trial involving an amputee. Two EMGs are attached to the stump for the dual-side control of the prosthesis with an elastic band. The vibromotor is fixed to the upper side of the stump with a second elastic band for the feedback restitution. The objects are placed within Hannes hand by the experimenter while the participant has closed eyes. The keyboard, placed in front of the participant, is used to indicate the grasped object stiffness by the user using the left hand.

The subjects were seated comfortably in front of a table (refer to Figure 4) with EMG sensors positioned on the forearm or stump using an elastic band. The electrodes measured the activity of the forearm muscles involved in the opening and closing of the hand (Flexor Carpi Ulnaris and Extensor Carpi Ulnaris, respectively), which were selected by manual inspection. The Hannes system was detached from the users' bodies (except for the two EMGs) and fixed on the table, lying between the subjects' arms with the palm up, to allow the experimenter place the objects to be grasped within the prosthetic hand. Hence, subjects were only asked to close and open their hand, not to approach or grasp the objects. The prosthesis was commanded in proportional-speed-control mode through the EMG signals. To convey the vibratory feedback, the vibromotor was positioned on the pisiform bone for able-bodied subjects and on the lateral epicondyle for the amputees by means of a second elastic band.

First, the minimum and maximum amplitude for the vibromotor was determined using the method of limits (Prins, 2016), to find the minimum level of perception and avoid discomfort. To this aim, the vibration intensity was increased in small steps (4–5% in the normalized scale of PWM). When the subject warned, as soon as it was perceptible, the sensing of a small and then of a strong sensation, the respective PWM was saved. Subsequently, 30% of the PWM range was adopted for soft objects and 100% was adopted for rigid objects. The vibration intensity was then modulated between these two values to generate clearly perceivable and localized vibrations that were not intrusive to the subject but intuitive for the encoding of the object stiffness.

Six objects (Figure 1) were randomly presented three times to the user by the experimenter and three void closures were also inserted along the test, to have a total number of 21 trials. Before the test phase, a training phase was performed to let the user become familiar with the feedback. A total of six closures were performed, alternating between rigid and soft objects without headphones and with open eyes, so the user could learn to associate the proper feedback with the right stiffness. Furthermore, the involved upper limb side was covered with a black blanket to strengthen a possible embodiment effect.

In the first phase to evaluate the classifier performance and the feedback effectiveness, the able-bodied subjects underwent a single test with a single condition. They performed the test with the 2FB condition. The participant was asked to wear headphones with white noise and to close the eyes (avoiding the sight of the prosthesis and the grasped object). The subject was not required to reach out to the object. Instead, the experimenter proceeded to insert it directly into the prosthesis, asking the subject to perform a full closure, and then to identify the stiffness of the squeezed object. The answer was provided by the subject's left hand pressing the keyboard arrows, left for rigid objects and right for soft objects. No button needed to be pressed when the prosthesis performed a void closure. Finally, the subject could reopen the eyes to check if the answer was correct.

In the second phase, a comparison between the four different feedback conditions, discussed in the “Feedback conditions” section, was carried out by five transradial amputees. The order of these four sessions was randomly presented to the amputees. Each condition had the same test protocol already described in the first phase with able-bodied subjects, in which the experimenter places the object inside the prosthetic hand and the amputee performs a grasp with closed eyes and gives the answer using the keyboard. At the end of each session, the proprioceptive drift was detected with respect to the initial arm position (refer to the “Amputees” section) and an *ad-hoc* questionnaire was administered (refer to Supplementary material).

### 2.5. Data analysis

All the outcomes and the evaluation methods used in this study were tested for normality using the Shapiro–Wilk test. A repeated measure one-way ANOVA or Friedman test was conducted depending on the outcome of the normality test (for the analysis of dataset with missing data, Skilling's Mack was applied in substitution of the Friedman test), while the multiple comparison test with Bonferroni correction was used for a *post-hoc* analysis. Mathworks MATLAB 2020b was used for the statistical analysis. The average of the measures used (error and efficiency) was computed for each subject and condition and compared across conditions. The threshold for statistical significance was set at p < 0.05. The results in the text are reported as mean and standard deviation.

#### 2.5.1. Able-bodied subjects

The primary outcome measure was the F1Score of the classifier on detecting the grasped object's stiffness expressed as a percentage, which takes into account the rate of false and true positives and false negatives (Powers, 2020). This result demonstrates that our approach to intrinsic sensor stiffness detection works properly. In addition, the F1Score was calculated on users' performance in recognizing objects' stiffness using the 2FB approach described in the “Feedback conditions” section. This latter was used to verify the usability and clarity of our feedback method.

#### 2.5.2. Amputees

The second phase involving five amputees was carried out to compare the four feedback conditions. To validate and demonstrate that the 2FB condition was effective and the best feedback restoration for the recognition of objects' stiffness, our hypothesis involving the following four evaluation methods was used: (i) F1Score of performance; (ii) reaction time to recognize the stiffness of the objects; (iii) proprioceptive drift; and (iv) *ad-hoc* questionnaire.

The F1Score of amputees' performance was calculated in all feedback conditions. Furthermore, the response time of each trial was also recorded for the four conditions. Low response times were considered positive results. For each amputee, the mean response time of each feedback condition was calculated to allow comparison.

As a quantitative measure of the embodiment, the proprioceptive drift toward the artificial limb was detected (Tsakiris and Haggard, 2005). Before covering the involved upper limb side with a black blanket, the initial position of the hand was marked with white tape. Immediately, after the experiment, the blanket was removed and the amputees were asked to close their eyes, raise their stump, and replace it in the perceived initial position. The lateral distance between the initial position and the one estimated after the trials was measured by the experimenter with a ruler in centimeters, together with the direction of the deviation (Barresi et al., 2021). Deviations toward the prosthesis were considered an effect of the embodiment process.

At the end of each session, amputees also had to complete a Likert-type 5-point questionnaire, providing a subjective evaluation. The questionnaire (refer to Supplementary material) aimed to assess subjectively the intuitiveness and comfortability of the feedback (seven questions), its utility for ADLs (three questions), and the embodiment (four questions). The possible answers ranged between 1 (strongly disagree) and 5 (strongly agree). Since all amputees performed the test in all conditions, the experimental design is within-subject.

## 3. Results

### 3.1. Able-bodied subjects

The classifier's average accuracy in identifying the object stiffness was tested on a total of 378 grasps (21 grasps × 18 subjects). Its average F1Score resulted to be 94.93% ± 3.94. The able-bodied subjects instead, due to the 2FB condition, reached an average F1Score of 94.08% ± 4.0 for the object's stiffness discrimination task.

**Figure 5:**
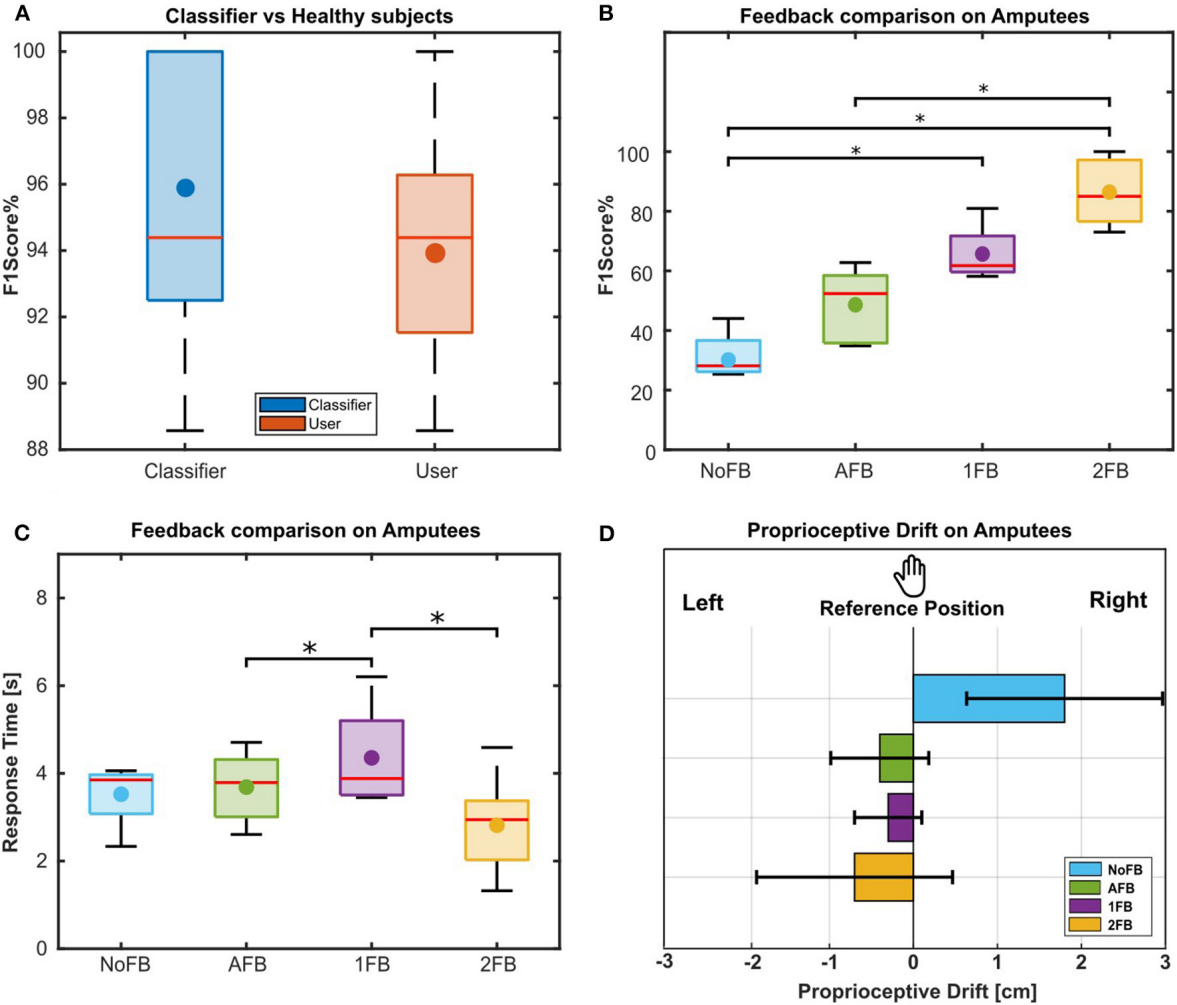
**(A)** F1Scores results of classifier with respect to able-bodied subjects. First, the F1Score of the classifier is calculated (blue box). Then, the F1Score of 18 able-bodied subjects is evaluated, based on the answer of classifier, in recognizing the objects' stiffness while receiving the 2FB condition (red box). A comparison between classifier's and subjects' F1Score was assessed and no significant difference was found between the two populations. **(B)** F1Scores results for amputees. F1Score obtained by amputees for each feedback condition. The Box chart shows the comparison between the distributions of the F1Scores obtained in each condition. The statistically significant difference is indicated by “^*^” (*p* < 0.05). **(C)** Response time results for amputees. Response time obtained by amputees for each experimental condition. The Box chart shows the comparison between the distributions of the response time obtained in each condition. The statistically significant difference is indicated by “^*^” (*p* < 0.05). **(D)** Proprioceptive drift of amputees for the different feedback conditions as a quantitative embodiment measure. The barplot shows the mean and standard deviation of the drift over amputees for each condition, the left direction is toward Hannes hand, and the right direction indicates a movement on the opposite side of Hannes hand. Deviations toward the prosthesis were considered an effect of the embodiment process.

Figure 5A shows the F1Score obtained by able-bodied subjects during the 2FB condition compared to the F1Score of the classifier performance. Since these data did not present a normal distribution, the Friedman test was applied to demonstrate that no statistical difference was detected between the two populations (*p* = 0.1).

### 3.2. Amputees

Figure 5B shows the boxplot of F1Score obtained by amputees for each of the four feedback conditions. It is possible to observe an ascending trend in the scores from the NoFB condition to the 2FB condition. In the NoFB condition, Amputee A3 data are missing because he found it impossible to accomplish the task without any feedback, stating that it was not possible to understand if the prosthesis was opened or closed. In the 1FB condition, Amputee A5 data are missing due to a recording problem.

For the NoFB condition, the F1Score among amputees is 31.41% ± 8.57, as indicated in Figure 5B with points, which is below the random chance probability of 33%. The statistically significant difference is indicated by “^*^” (*p* < 0.05). Only Amputee A1 achieved a higher F1Score with respect to random chance (F1Score = 44.03%). The distributions resulted to be normal, so the statistical analysis applied was the ANOVA. As shown in Figure 5B, the 2FB condition presents a statistically significant difference with respect to the NoFB (*p* < 0.001) and AFB (*p* < 0.001) conditions. Furthermore, the 1FB condition is statistically different from the NoFB condition (*p* = 0.0031). The average F1Score calculated from the five amputees' responses during the 2FB experimental session is 86.41% ± 11.6.

Figure 5C shows the average response time for amputees in each feedback condition, in which the statistically significant difference is indicated by “^*^” (*p* < 0.05). All amputees, except A1, achieved the lowest response time during the 2FB condition (2.82 s ± 1.2), which also produced the best results in terms of F1Scores. A statistical analysis was performed between the different conditions. The distribution resulted to be not normal and, given the presence of some missing data, the Skillings–Mack test was applied. As shown in Figure 5C, there is a statistically significant difference between AFB and 1FB conditions (*p* = 0.02) and between 1FB and 2FB conditions (*p* = 0.04).

The mean proprioceptive drift for each feedback condition was calculated, and it is reported in Figure 5D as bar plots with standard deviations. On average, the five amputees estimated the position of their right arm after the experiment as 1.8 cm ± 1.17 right (in the opposite side of the prosthesis) during the NoFB condition while 0.4 cm ± 0.58, 0.3 cm ± 0.4, and 0.7 cm ± 1.17 toward left and hence Hannes during AFB, 1FB, and 2FB conditions, respectively. The only significant difference was found between NoFB and 2FB conditions (*p* = 0.017) with the Nemenyi test for a *post-hoc* comparison.

According to comparisons performed through the Friedman test (because the scales are discrete and the actual data do not match the assumptions for other inferential techniques), three scales of the questionnaire showed significant effects of the feedback conditions. The subjective evaluations collected about the sessions show that in the 2FB condition:

- Scale 1 made it significantly easier to perceive the difference between soft and hard objects (*p* = 0.027);
- Scale 2 was significantly more intuitive for soft objects (*p* = 0.015);
- Scale 3 was significantly intuitive for rigid objects (*p* = 0.005).

According to Scale 3 scores, the *post-hoc* comparisons performed through the Nemenyi test show how the proposed feedback condition was significantly more intuitive for rigid objects than the NoFB condition (*p* = 0.025) and 1FB condition (*p* = 0.017). Furthermore, the Friedman test showed a significant effect of the 2FB condition (*p* = 0.019), especially considering the results of the Nemenyi test for the *post-hoc* comparison between 2FB and NoFB conditions (*p* = 0.017).

## 4. Discussion

The present study explored the possibility to recognize objects' stiffness with an under-sensorized prosthesis. The reference position, the encoder position, and the motor-side current available on Hannes were used to feed a pattern recognition algorithm, capable of generating different vibratory feedback to allow the subject to decode the relative objects' stiffness.

The F1Score of the classifier during the 2FB condition tested with able-bodied subjects (Figure 5A) was very high (94.93% ± 3.94), demonstrating that the only sensors available on Hannes (motor-side current and encoder position) provide sufficient information for an object stiffness discrimination task using an NLR algorithm. However, it is necessary to consider that the classifier, during this experiment, was only tested on six objects of different shapes but with almost the same dimensions (chosen to replicate the objects used during the Cybathlon race, Caserta et al., 2022). Furthermore, the F1Score obtained by able-bodied subjects (Figure 5A) on discriminating the object stiffness was also very good, proving the usability and efficacy of this feedback approach on a user case.

The positive results of the first phase allowed us to evaluate the object stiffness recognition approach on five transradial amputees. In this second phase, we tested four approaches of the feedback scheme (Figure 5B). In the NoFB condition, we expected a correct identification of the right stiffness around the random chance probability (33%). Actually, the F1Score for the NoFB condition was even lower than this percentage (F1Score = 31.41% ± 8.57), as amputees stated they were forced to guess since being deprived of any possible clue. The AFB condition presents a higher average F1Score (48.62% ± 12.56) with respect to the NoFB one, indicating that the motor noise provides less help in this kind of task. This is true for expert users like Amputee A1, who reached the highest score (62.78%), while it is less evident from others like Amputees A3 (34.85%) and A5 (36.11%), who scored almost as random chance. Differently, in the 1FB condition, almost everyone improved their performance (F1Score = 65.67% ± 10.34) with respect to NoFB and AFB conditions. In this condition, the users were clearly helped in recognizing the void closures, since those were the only ones without vibratory feedback. Moreover, most of the amputees declared that even if the intensity of the vibration was the same for rigid and soft objects, they were able to perceive a difference based on the vibration onset. Since soft objects are more compliant, the motor-side current takes more time to rise with respect to a rigid object. Hence, the vibration is slightly late. For this reason, the 1FB condition resulted to be statistically better than the NoFB one, unlike the AFB condition which has no significant difference with respect to the NoFB condition. Overall, the 2FB condition provided the best results (F1Score 86.41% ± 11.6), demonstrating to be significantly more helpful with respect to the other conditions and indicating that the difference in vibration, correspondent to the rigid and soft objects, was sufficiently distinguishable by the users, as we expected. This proves the advantages that this type of feedback can provide to prosthesis users as additional information to the incidental feedback (i.e., auditory feedback).

The reduction in the response time (Figure 5C) in the 2FB condition (2.82 s ± 1.2) is another proof of the efficiency of the implemented distinct vibratory feedback, meaning the amputees needed a short time to understand object's stiffness and enhancing the intuitiveness of the method. This parameter is significantly lower in 2FB (2.82 s ± 1.2) condition with respect to the NoFB (3.52 s ± 0.8), AFB (3.7 s ± 0.83), and 1FB (4.35 s ± 1.28) ones, suggesting that in these latter, the amputees needed to put quite an effort in discriminating between the objects instead.

The proprioceptive drift (Figure 5D) shows an effect of the feedback on the embodiment, especially according to the comparison between 2FB (0.7 cm ± 1.17 toward Hannes hand) and NoFB (1.8 cm ± 1.17 opposite to Hannes hand) conditions. Interestingly, the results could indicate that the presence of a source of feedback is important for summoning the embodiment process. Precisely, the highest impact on the proprioceptive drift was found with the 2FB condition, suggesting that this specific vibratory feedback was the most effective one during the embodiment process. However, a larger sample size is necessary to check potentially higher effects caused by the 2FB condition.

Three scales in the subjective questionnaire significantly highlight the benefits offered by the stimulations provided in the 2FB condition as intuitive feedback, especially for rigid objects. This indicates a possible effect of the feedback on the embodiment (refer to Figure 5D). However, a larger sample is necessary to deepen our understanding of the potential effects of the 2FB condition on embodiment measures in dedicated experiments. Overall, and regardless of the statistical significance, the results seem to point out the superiority of the 2FB condition over all aspects of user experience considered in this study. The qualitative observations provided by the amputees need a larger sample to extract potential user requirements.

## 5. Conclusion

This study presents the implementation of an online, i.e., real-time, dedicated stiffness detection strategy to provide grasp-oriented vibratory feedback using the Hannes prosthetic hand in a closed-loop scenario. As a further progression of our previous studies, in which we exploited a virtual simulation to find the intrinsic variables correlated to the grasped object's stiffness, this study builds upon those preliminary findings and presents a refined and improved methodology, its implementation, and its clinical validation. The main aim was to implement an online strategy exploiting such measurements (motor-side current, encoder position, and reference position) to detect the stiffness of real objects (without increasing the system complexity with *ad-hoc* force sensing) and to validate such strategy with a first preliminary study with end-users.

The classifier was tested by 18 able-bodied subjects on six objects and resulted to be sufficiently accurate in discriminating between void, soft, and rigid grasps. The stiffness information was conveyed to the users through a single vibromotor, whose intensity changed based on the grasp type, i.e., high intensity for rigid objects and low intensity for soft objects in our proposed feedback condition (2FB condition). This feedback modality was compared to three other control conditions (NoFB, AFB, and 1FB) in a user study involving five mono-lateral amputees. Results showed a statistically significant improvement in users' performances both in terms of F1Score and response time for the 2FB condition. Moreover, this condition was appreciated by the users, as demonstrated by the subjective questionnaires, which highlighted its intuitiveness, comfortability, and usefulness. This result was also confirmed by the analysis of the proprioceptive drift, which showed an improvement in the prosthesis embodiment. Hence, we can state that our proposed feedback modality was the best among those tested.

In the future, the classifier should be tested on a higher variety of objects with different dimensions and stiffness, especially to investigate the influence of the dimension on the algorithm's performance. Reach and grasp tasks, with active usage of prosthesis, will be implemented to provide a more realistic validation of the usability and effectiveness of our solution within ADL and real scenarios. A higher number of prosthesis users will be involved to better assess the effect of the feedback on the embodiment and its appreciation.

The present study can have a relevant impact on the application of intrinsic sensor detection of object stiffness, as it points out that this object recognition strategy and vibrotactile feedback restitution on upper limb prosthesis could be effectively used as an intuitive and effective closed-loop daily living solution. Such a solution could facilitate the identification of a precise and delicate grasp rather than a strong and powerful one during different object manipulations.

## Data availability statement

The raw data supporting the conclusions of this article will be made available by the authors, without undue reservation.

## Ethics statement

The studies involving human participants were reviewed and approved by AVEC Area Vasta Emilia Centro Protocol Code: CP-PPRAS1/1-03. The patients/participants provided their written informed consent to participate in this study.

## Author contributions

GiuB, AM, NB, GiaB, and GC conceived the study. GiuB and GC administered the experiments. GiuB, AM, NB, and GC designed the figures. AM prepared the figures. All the authors contributed to the writing and reading and approved the final version of the manuscript.

## Footnotes

^1^Vybronics is a datasheet of a product available in the following link: https://www.vybronics.com/wp-content/uploads/datasheet-files/Vybronics-VC0625B001L-Jinlong-C0625B001L-datasheet.pdf.
