# Supplementary material for: Object stiffness recognition and vibratory feedback without ad-hoc sensing on the Hannes prosthesis: A machine learning approach
Source: Front Neurosci. 2023 Feb 16;17:1078846. doi: 10.3389/fnins.2023.1078846 (PMC9978002; doi:10.3389/fnins.2023.1078846)
Supplement: Supplementary file 1 [file Data_Sheet_1.docx]

Supplementary Material

# Hannes hand and control implementation

**
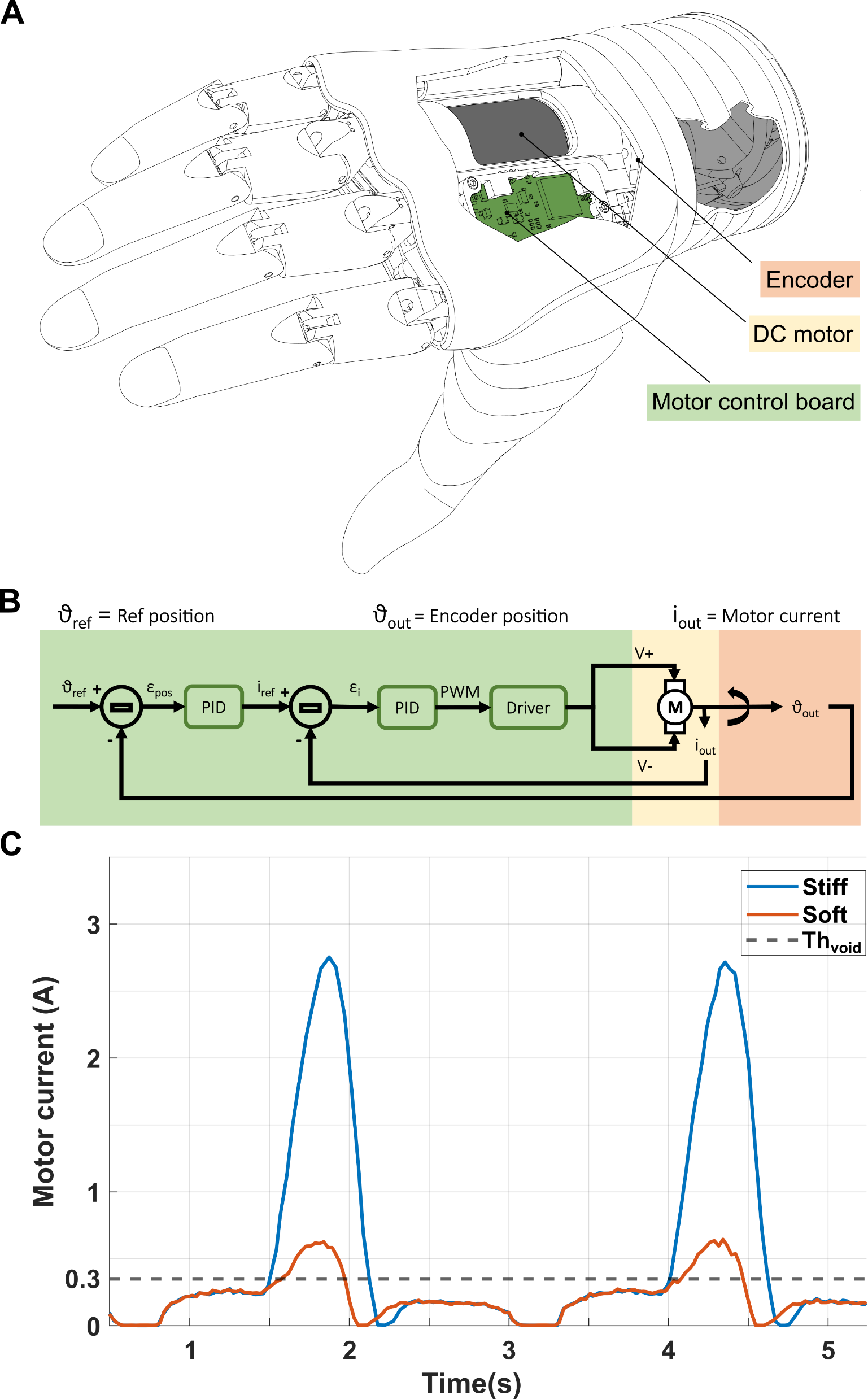
**

**Figure 1** Hannes system. A: The Hannes prosthetic hand with its principal electrical components. B: The control block scheme to move the prosthesis and record intrinsic signals. C: The motor current recorded during the grasp of rigid or soft objects. The horizontal gray line represents the current threshold to determine if the hand is grasping object or it is moving free.

# Ad-hoc Questionnaire

| Questions | | |
| --- | --- | --- |
| Feedback scheme | **1** | It was easy to sense the difference between the two feedback for soft and rigid objects |
| Feedback scheme | **2** | The feedback for soft objects was intuitive, clear and easy to understand |
| Feedback scheme | **3** | The feedback for rigid objects was intuitive, clear and easy to understand |
| Embodiment | **4** | It seemed like I was causing the grasp made by the prosthesis |
| Feedback scheme | **5** | Which was the most intuitive feedback? Why? |
| Feedback scheme | **6** | The feedback for soft objects was comfortable and not annoying at all |
| Embodiment | **7** | It felt like the prosthesis was part of my body |
| Feedback scheme | **8** | The feedback for rigid objects was comfortable and not annoying at all |
| Feedback scheme | **9** | Which feedback was the most comfortable? Why? |
| Embodiment | **10** | It felt that the tactile sensation was coming from somewhere between my arm and the prosthesis |
| Activities of Daily Living | **11** | This kind of feedback can be very useful in the everyday life |
| Activities of Daily Living | **12** | This feedback would make my everyday activities safer |
| Embodiment | **13** | It seemed that the tactile sensation I felt was caused directly from the object in the prosthesis |
| Activities of Daily Living | **14** | This feedback would make my everyday activities more efficient |

# Amputees’ answers to the questionnaire

|  | ***Questions*** | ***NoFB*** | ***AFB*** | ***1FB*** | ***2FB*** |
| --- | --- | --- | --- | --- | --- |
| *A1* | **1** | 4 | 3 | 2 | 4 |
|  | **2** | 4 | 2 | 1 | 5 |
|  | **3** | 3 | 3 | 2 | 5 |
|  | **4** | 4 | 4 | 3 | 5 |
|  | **5** | Soft, for the used time | - | A little, since the vibration was the same | It seemed to feel the grasp |
|  | **6** | 4 | 4 | 3 | 4 |
|  | **7** | 4 | 4 | 4 | 4 |
|  | **8** | 3 | 4 | 3 | 4 |
|  | **9** | - | - | Soft | Soft object had low vibration |
|  | **10** | 4 | 4 | 3 | 3 |
|  | **11** | 4 | 4 | 4 | 4 |
|  | **12** | 4 | 4 | 4 | 4 |
|  | **13** | 3 | 3 | 3 | 4 |
|  | **14** | 5 | 4 | 3 | 4 |

|  | ***Questions*** | ***NoFB*** | ***AFB*** | ***1FB*** | ***2FB*** |
| --- | --- | --- | --- | --- | --- |
| *A2* | **1** | 1 | 3 | 3 | 5 |
|  | **2** | 1 | 1 | 4 | 5 |
|  | **3** | 1 | 4 | 2 | 5 |
|  | **4** | 1 | 3 | 5 | 4 |
|  | **5** | No object | Rigid, distinct noise | Blue ball, specific vibration | All |
|  | **6** | 2 | 4 | 5 | 5 |
|  | **7** | 1 | 3 | 5 | 4 |
|  | **8** | 2 | 4 | 5 | 5 |
|  | **9** | No object | Rigid | Soft | All |
|  | **10** | 1 | 3 | 3 | 3 |
|  | **11** | 1 | 4 | 4 | 5 |
|  | **12** | 1 | 4 | 3 | 4 |
|  | **13** | 1 | 2 | 3 | 4 |
|  | **14** | 1 | 4 | 3 | 4 |

|  | ***Questions*** | ***NoFB*** | ***AFB*** | ***1FB*** | ***2FB*** |
| --- | --- | --- | --- | --- | --- |
| *A3* | **1** | 1 | 1 | 1 | 5 |
|  | **2** | 1 | 1 | 1 | 5 |
|  | **3** | 1 | 1 | 1 | 5 |
|  | **4** | 3 | 2 | 3 | 5 |
|  | **5** | Nothing | Nothing |  | Both |
|  | **6** | - | 4 | 5 | 5 |
|  | **7** | 1 | 1 | 1 | 2 |
|  | **8** | - | 4 | 5 | 5 |
|  | **9** | Nothing | Nothing | Nothing | Both |
|  | **10** | 1 | 4 | 3 | 3 |
|  | **11** | 1 | 1 | 2 | 5 |
|  | **12** | 1 | 1 | 2 | 5 |
|  | **13** | 1 | 1 | 4 | 2 |
|  | **14** | 1 | 1 | 2 | 5 |

|  | ***Questions*** | ***NoFB*** | ***AFB*** | ***1FB*** | ***2FB*** |
| --- | --- | --- | --- | --- | --- |
| *A4* | **1** | 1 | 2 | 1 | 4 |
|  | **2** | 1 | 2 | 1 | 4 |
|  | **3** | 1 | 2 | 1 | 4 |
|  | **4** | 1 | 3 | 2 | 3 |
|  | **5** | - | Void because the motor made sound for longer time | Void | Same |
|  | **6** | 1 | 5 | 5 | 5 |
|  | **7** | 1 | 4 | 2 | 4 |
|  | **8** | 1 | 5 | 5 | 5 |
|  | **9** | - | All the same | Same | Same |
|  | **10** | 1 | 2 | 2 | 4 |
|  | **11** | 1 | 3 | 1 | 4 |
|  | **12** | 1 | 3 | 1 | 4 |
|  | **13** | 1 | 3 | 1 | 3 |
|  | **14** | 1 | 2 | 1 | 4 |

|  | ***Questions*** | ***NoFB*** | ***AFB*** | ***1FB*** | ***2FB*** |
| --- | --- | --- | --- | --- | --- |
| *A5* | **1** | 2 | 1 | 3 | 5 |
|  | **2** | 3 | 3 | 4 | 5 |
|  | **3** | 2 | 3 | 2 | 4 |
|  | **4** | 3 | 4 | 5 | 5 |
|  | **5** | Soft from the muscle contraction | Soft from the noise of the grasp | Soft for longer vibration | Soft because it’s lighter and it gives the idea of a softer object |
|  | **6** | 5 | 5 | 5 | 5 |
|  | **7** | 4 | 5 | 5 | 5 |
|  | **8** | 5 | 5 | 5 | 5 |
|  | **9** | Soft, with the contraction you can understand the type of object | Both | Same | Same |
|  | **10** | 3 | 5 | 1 | 1 |
|  | **11** | 5 | 4 | 4 | 5 |
|  | **12** | 5 | 5 | 5 | 5 |
|  | **13** | 5 | 5 | 5 | 5 |
|  | **14** | 5 | 5 | 5 | 5 |
